# Specific Variants in the MLH1 Gene Region May Drive DNA Methylation, Loss of Protein Expression, and MSI-H Colorectal Cancer

**DOI:** 10.1371/journal.pone.0013314

**Published:** 2010-10-13

**Authors:** Miralem Mrkonjic, Nicole M. Roslin, Celia M. Greenwood, Stavroula Raptis, Aaron Pollett, Peter W. Laird, Vaijayanti V. Pethe, Theodore Chiang, Darshana Daftary, Elizabeth Dicks, Stephen N. Thibodeau, Steven Gallinger, Patrick S. Parfrey, H. Banfield Younghusband, John D. Potter, Thomas J. Hudson, John R. McLaughlin, Roger C. Green, Brent W. Zanke, Polly A. Newcomb, Andrew D. Paterson, Bharati Bapat

**Affiliations:** 1 Department of Laboratory Medicine and Pathobiology, University of Toronto, Toronto, Ontario, Canada; 2 Samuel Lunenfeld Research Institute, Mount Sinai Hospital, Toronto, Ontario, Canada; 3 Department of Pathology and Laboratory Medicine, Mount Sinai Hospital, Toronto, Ontario, Canada; 4 Program in Genetics and Genome Biology, Hospital for Sick Children, Toronto, Ontario, Canada; 5 Dalla Lana School of Public Health, University of Toronto, Toronto, Ontario, Canada; 6 University of Southern California Epigenome Center, University of Southern California, Los Angeles, California, United States of America; 7 Ontario Familial Colorectal Cancer Registry, Cancer Care Ontario, Toronto, Ontario, Canada; 8 Clinical Epidemiology Unit, Memorial University of Newfoundland, St. John's, Newfoundland, Canada; 9 Department of Laboratory Medicine and Pathology, Mayo Clinic, Rochester, Minnesota, United States of America; 10 Department of Surgery, University of Toronto, Toronto, Ontario, Canada; 11 Discipline of Genetics, Memorial University of Newfoundland, St. John's, Newfoundland, Canada; 12 Public Health Sciences Division, Fred Hutchinson Cancer Research Centre, Seattle, Washington, United States of America; 13 Ontario Institute for Cancer Research, Toronto, Ontario, Canada; 14 Department of Molecular Genetics, University of Toronto, Toronto, Ontario, Canada; 15 Department of Medical Biophysics, University of Toronto, Toronto, Ontario, Canada; 16 Ottawa Hospital Research Institute, Ottawa, Ontario, Canada; University of Barcelona, Spain

## Abstract

**Background:**

We previously identified an association between a mismatch repair gene, *MLH1*, promoter SNP (rs1800734) and microsatellite unstable (MSI-H) colorectal cancers (CRCs) in two samples. The current study expanded on this finding as we explored the genetic basis of DNA methylation in this region of chromosome 3. We hypothesized that specific polymorphisms in the *MLH1* gene region predispose it to DNA methylation, resulting in the loss of *MLH1* gene expression, mismatch-repair function, and consequently to genome-wide microsatellite instability.

**Methodology/Principal Findings:**

We first tested our hypothesis in one sample from Ontario (901 cases, 1,097 controls) and replicated major findings in two additional samples from Newfoundland and Labrador (479 cases, 336 controls) and from Seattle (591 cases, 629 controls). Logistic regression was used to test for association between SNPs in the region of *MLH1* and CRC, MSI-H CRC, *MLH1* gene expression in CRC, and DNA methylation in CRC. The association between rs1800734 and MSI-H CRCs, previously reported in Ontario and Newfoundland, was replicated in the Seattle sample. Two additional SNPs, in strong linkage disequilibrium with rs1800734, showed strong associations with *MLH1* promoter methylation, loss of MLH1 protein, and MSI-H CRC in all three samples. The logistic regression model of MSI-H CRC that included *MLH1*-promoter-methylation status and MLH1 immunohisotchemistry status fit most parsimoniously in all three samples combined. When rs1800734 was added to this model, its effect was not statistically significant (*P*-value  = 0.72 vs. 2.3×10^−4^ when the SNP was examined alone).

**Conclusions/Significance:**

The observed association of rs1800734 with MSI-H CRC occurs through its effect on the *MLH1* promoter methylation, MLH1 IHC deficiency, or both.

## Introduction

Colorectal cancer (CRC) is the fourth most common cancer, and second leading cause of cancer-related deaths in North America [1]. CRCs can be parsimoniously subdivided into two major groups defined by the genetic pathways involved. The suppressor pathway, observed in >80% of CRC cases, involves abnormalities of the APC/wingless signalling pathway and is characterized by frequent somatic mutations of oncogenes and loss of heterozygosity of tumor suppressor genes, chromosomal instability, and microsatellite stable (MSS) tumors. The mutator pathway, on the other hand, accounts for ∼15–20% of CRC cases and results from a deficiency of the mismatch-repair (MMR) system, which leads to genome-wide microsatellite instability (MSI) [2], [3]. MSI tumors have clinicopathologic features distinct from MSS tumors in that they tend to occur more commonly in proximal colon, have mucinous histology, tumor infiltrating lymphocytes, poor differentiation, and Crohn's-like reaction [4]. CRCs can also be classified based on epigenetic instability into CpG Island Methylator Phenotype (CIMP)-positive and CIMP-negative tumors [5]. CIMP-positive CRC tumors can be subsequently subdivided into two groups, a more common CIMP1 tumors, which are MSI-H due to *MLH1* promoter methylation, and CIMP2 tumors, which are MSS [5]. Approximately 80–90% of sporadic MSI CRCs exhibit loss of MMR function due to *MLH1* promoter methylation [6], [7]. The potential mechanism by which *MLH1* is epigenetically silenced is unclear.

Our previous work aimed to elucidate the role of a panel of SNPs in MMR genes in CRC. Included in this panel was the *MLH1*-93G>A promoter polymorphism (rs1800734), and we observed its association with MSI-H tumors in two samples from the Canadian provinces of Ontario and Newfoundland and Labrador [8]. Several studies subsequently confirmed and expanded on our findings and have observed associations between the *MLH1*-93G>A polymorphism and *MLH1* promoter methylation in CIMP CRCs, as well as MLH1 IHC deficiency [9], [10], [11]. However, no predictive model has been proposed to describe such findings. The association between the *MLH1* promoter polymorphism (rs1800734) and methylation may indicate sequence specificity to DNA methylation.

**Figure 1:**
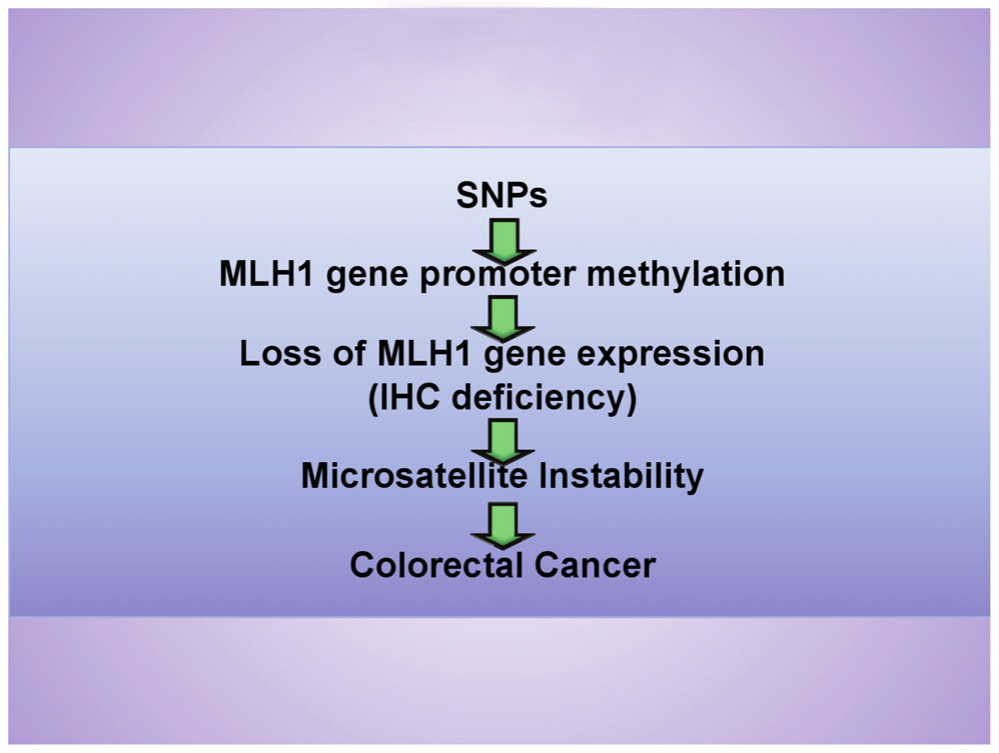
Proposed model for genetic susceptibility to DNA methylation in sporadic MSI-H CRCs. Specific SNPs predispose the region, including the *MLH1* gene promoter, to methylation, which results in promoter silencing and loss of *MLH1* gene expression that is measured by immunohistochemical staining. Loss of the *MLH1* gene expression leads to genome-wide microsatellite instability and MSI-H colorectal cancer.

We hypothesized a stepwise progression to MSI-H CRCs based on genetic susceptibility to DNA methylation leading to *MLH1* gene silencing and microsatellite instability (**Figure 1**). Further, we hypothesized that the *MLH1*-93G>A polymorphism may be in strong linkage disequilibrium (LD) with other variants, and that one or more of these variants predispose the region to methylation, which then results in loss of *MLH1* gene expression and a defective MMR system, leading to microsatellite instability. We have undertaken a population-based approach using three independent samples. This study used a unique combination of genetic epidemiology and functional strategies to identify and characterize alleles that play a role in modifying CRC development in an important and common subgroup of cases.

## Materials and Methods

### SNP Selection Criteria

The polymorphisms analyzed by 5′ nuclease assay in this study were selected on the basis of extensive database and literature searches as described previously [8], [12]. The 500 kb region of chromosome 3 surrounding *MLH1* was genotyped for all available polymorphisms from a combination of Affymetrix GeneChip Human Mapping 100K and 500K platforms. In addition, we selected SNPs in the region of interest that are in strong LD with rs1800734 in the HapMap data (release 27 in CEU population), publicly available at http://www.hapmap.org. Two such SNPs were identified and were also included.

### Study Subjects

We conducted this study with subjects from three different locations: the province of Ontario, the province of Newfoundland and Labrador (hereafter referred to as Newfoundland), and the Seattle metropolitan area. In all locations, only individuals with a single tumor were included. CRC patients and unaffected controls from Ontario and Newfoundland were accrued as described previously [8], [12]. Briefly, for Ontario 1004 CRC patients and 1957 controls were identified by the Ontario Familial Colorectal Cancer Registry (OFCCR) [13]. In order to minimize the potential for population stratification we excluded from the analyses cases who were non-white and those who did not report ethnicity. Of the 1004 case patients, 929 were white. No related cases were used in the study. Further, we excluded all CRC patients with known MMR germline gene mutations (11 cases with a known mutation in MLH1, 10 in MSH2, and one in MSH6) and all CRC cases that were deficient in one of the MMR proteins, other than MLH1 (14 MSH2/MSH6 IHC deficient tumors). 901 CRC patients remained and constitute the Ontario cases. All patient information as well as blood and tissue specimens were obtained as described previously [8].

A total of 1957 control subjects from Ontario agreed to participate in the study and completed all three questionnaires (family, personal, and diet questionnaires). Of the 1957, 1314 controls provided blood samples, and 1097 of them were white. These 1097 control subjects were successfully genotyped and thus constituted the Ontario controls. Approximately 21% of OFCCR cases and 12% of controls have first-degree relatives affected with CRC.

The accrual pattern followed by the Newfoundland Familial Colorectal Cancer Registry (NFCCR) was similar to that followed by the OFCCR. Patients with CRC were identified through the Newfoundland tumor registry; 1144 CRC patients were identified, of whom 747 responded to the family history questionnaire and 555 provided blood samples; 490 provided ethnicity information and were classified as white. No related cases were used in the study. Four CRC cases with known germline mutations in MSH2 were excluded, as were 11 non-MLH1 MMR IHC deficient cases (5 for MSH2, 5 for MSH6, and 1 for PMS2 deficiency). The remaining 479 CRC patients constitute the Newfoundland cases.

Newfoundland controls were recruited using random digit dialing, and matched to cases by sex and 5-year age group; 1602 controls agreed to participate, of whom 336, to this stage, have completed all three questionnaires and provided blood samples. No related controls were used in the study. Approximately 31% of NFCCR cases and 18% of controls had first-degree relatives affected with CRC.

For Seattle, cases and controls were recruited by the Fred Hutchinson Cancer Research Center (FHCRC) as described previously [14]. Briefly, CRC patients who were diagnosed between the ages of 20 and 74 years in Washington's King, Snohomish, or Pierce Counties between January 1998 and June 2002 were contacted. All CRC cases were included regardless of family history. Of the 1814 cases and 1531 controls who completed the two questionnaires, 1497 cases and 745 controls donated a blood sample. For this study, we obtained DNA samples for 668 CRC cases and 667 controls of Caucasian ethnicity. Fifteen MMR IHC deficient CRC cases were excluded (10 for MSH2, 1 for MSH6, and 4 for PMS2 deficiency). No related cases or controls were used in the study. Approximately 14% of FHCRC cases and 8% of controls had first-degree relatives affected with CRC.

Data were collected on age at diagnosis (for cases), age at completion of the family history questionnaire, tumor location, tumor stage, and tumor grade, when available, through review of pathologic and/or surgical reports. Tumors were staged and graded according to the method of the American Joint Committee on Cancer [15]. Blood and tissue specimens were obtained upon informed written consent to participate in the study, as per protocols approved by the research ethics boards of Mount Sinai Hospital, University of Toronto, Memorial University of Newfoundland, and Fred Hutchinson Cancer Research Center.

### Molecular Genetic Analysis

#### Single-Nucleotide Polymorphism Genotyping

Peripheral blood lymphocytes were isolated from whole blood by use of Ficoll–Paque gradient centrifugation according to the manufacturer's protocol (Amersham Biosciences, Baie d'Urfé, Quebec, Canada). Phenol–chloroform or the Qiagen DNA extraction kit (Qiagen Inc., Montgomery Co., MD) was used to extract genomic DNA from lymphocytes. The fluorogenic 5′ nuclease polymerase chain reaction assay or the TaqMan assay [16] was used to genotype each of the following five SNPs: *MLH1*–93G>A (rs1800734), I219V (rs1799977), IVS14-19A>G (rs9876116), *LRRFIP2* intron 26 IVS26-18T>C (rs749072), *LBA1* intron 8 (rs4431050), and intergenic rs13098279. Sequences of primers and probes as well as the master reaction mixtures for rs1800734, rs1799977, and rs9876116 were described previously [8]. The *LRRFIP2* rs749072, *LBA1* rs4431050, and intergenic rs13098279 polymorphisms were genotyped by use of the Eurogentec qtPCR kit (Eurogentec, San Diego, CA) [8]. Sequences of primers and probes for rs749072, rs13098279, and rs4431050 are provided in **Supplementary File S4**.

SNPs located in the 500 kb region of chromosome 3 surrounding the *MLH1* gene were genotyped in the Ontario samples using the Affymetrix GeneChip Human Mapping 100K and 500K platforms as a part of the Assessment of Risk of Colorectal Tumors in Canada (ARCTIC) project, described previously [17]. 94 SNPs in the 500 kb region, in addition to the 5 SNPs genotyped by TaqMan, were genotyped for the Ontario samples spanning the following genes: *DCLK3*, *LBA1*, *EPM2AIP1*, *MLH1*, *LRRFIP2*, and *GOLGA4*. The list of SNPs genotyped for the Ontario samples is provided in **Supplementary** **File S1**. The Newfoundland and Seattle samples were genotyped using the Illumina ISelect 500K Chip platform. A total of 16 SNPs in this region were genotyped including rs1800734, rs749072, and rs13098279. The Newfoundland samples were further characterized for three polymorphisms: rs1799977 and rs9876116 genotyped previously [8], and *LBA1* rs4431050. The rs1800734 SNP was genotyped both by the Affymetrix Chips and Taqman platforms in Ontario and was used to validate genotyping calls. Out of 1884 samples genotyped by both methods there were 11 discordant calls (0.58%, **Supplementary File S1**).

The quality control for genotyping was performed as described previously [17]. Briefly, SNPs were excluded from the data analysis if the minor allele frequency was less than 1% and the call rate was less than 87% in the controls in each of the three study centres. Additionally, SNPs were excluded if the *P*-value from a test for Hardy-Weinberg equilibrium was less than 10^−4^ in the controls. Individuals were excluded if the genotyping call rate was less than 87%.

#### Tumor Microsatellite Instability Analysis

Tumor MSI analysis was performed as described previously [18]. Briefly, paraffin-embedded colorectal tumor and matched normal colonic tissue from patients with incident cases of CRC were microdissected in areas with more than 70% cellularity. PCR on DNA from CRC tumor and matched normal colonic tissue was used to establish and compare the MSI patterns. MSI analysis was carried out with at least five microsatellite markers from the panel of 10 microsatellite markers, as recommended by the National Cancer Institute [19]. MSI status was assigned as MSI high (MSI-H, ≥30% unstable markers among all markers tested), MSI low (MSI-L, <30% markers unstable), or microsatellite stable (MSS, no unstable markers) as recommended [19]. For the analysis, MSI-L and MSS groups were combined into one group (hereafter referred to as “MSS/L”). Primers were obtained from Applied Biosystems (Foster City, CA), and primer sequences were described previously [8].

### MMR Protein Immunohistochemical Staining

Formalin-fixed, paraffin-embedded CRC tissues, collected for diagnostic purpose, sectioned at 4 µm were deparaffinized and rehydrated with alcohol and xylene for immunohistochemical analysis of MLH1 as described previously [20], [21]. Following rehydration, the slides were placed into either a pressure cooker or microwave antigen retrieval medium (10mmol/L citrate buffer at pH 6.0 for 3 minutes at 115°C in microMED T/T Mega; Hacker Instruments & Industries, Inc., Fairfield, NJ). Protein blocker (20%) with avidin was used to prevent nonspecific binding (Signet Laboratories, Inc, Dedham, MA). After the slides were washed in PBS, the sections were incubated with mouse antibody against MLH1 (1∶40; G168-728, PharMingen, San Diego, CA), MSH2 (1∶100; FE 11, Oncogene Research Products, Cambridge, MA), MSH6 (1∶100; 44, BD Transduction Laboratories, Mississauga, Ontario, Canada), or PMS2 (1∶50; BD Biosciences PharMingen, Mississauga, Ontario, Canada) for 1 hour. The antibodies were then detected using avidin-biotin: 3,3′-diaminobenzidine tetrachloride was used as the chromogen and hematoxylin for counterstaining.

### MLH1 Promoter Methylation Analysis

*MLH1* promoter methylation was analyzed using MethyLight [22], [23]. Tumor DNA from the available cases was subject to sodium bisulfite conversion using EZ DNA Methylation Gold Kit (Zymo Research, Orange, CA) per manufacturer's recommendations.

MethyLight analysis of the *MLH1* promoter was performed as previously described [23]. The Alu-C4 control reaction was used to normalize for bisulfite-converted input DNA [23]. The samples were classified as positive for *MLH1* promoter methylation if percent methylated reference (PMR) ≥10, as described previously [23]. The primer and probe sequences for the *MLH1* and Alu-C4, as well as the real-time PCR program for MethyLight analysis have been previously reported [23]. All assays were run in 96-well polypropylene plates (Axygen Scientific, Union City, CA) and the results were analyzed using the ABI 7500HT Real-Time PCR instrument and the accompanying software, SDS version 2.2 (Applied Biosystems, Foster City, CA). Independent quality control for the MLH1 promoter methylation analysis was performed externally on 15% of Ontario samples.

### Statistical Analysis

Each of the following outcomes was tested for association with each SNP using logistic regression: colon cancer (all CRC cases versus controls), methylation (*MLH1* methylated tumors versus non-methylated tumors), IHC (MLH1 IHC-deficient versus proficient tumors), and MSI (MSI-H versus MSS/L tumors), using an additive coding of genotypes for each SNP. Sex and age at exam, collected for patients and unaffected controls, were used as covariates when CRC was the outcome, whereas sex and age at diagnosis (available for patients only) were used in models with the other outcomes. Analysis of separate models for the three collection sites and the combined dataset was undertaken. In the analysis of the combined data, site was included as a covariate.

Multiple logistic regression models for MSI status were also evaluated in the subset of the data in which there were no missing values for all of the variables included in the models (age, MSI, IHC, methylation, and three SNPs). MSI status was regressed on combinations of IHC, methylation and SNP, for each of three SNPs that showed associations in the initial logistic regression models. Since the sample sizes are small, particularly in Seattle, the regression was performed with all samples combined, while using a covariate for recruitment location. To check for consistency in the results, the models were also run on each sample separately. Due to the strong association between MSI, IHC, and methylation and nearly complete separation, maximum penalized likelihood was used to produce finite parameter estimates [24]. All statistical analyses were performed with R 2.7.0 (http://www.R-project.org).

In order to control for the effect of multiple testing, an effective number of tests was estimated for Ontario, Seattle and Newfoundland, based on the procedure of Li and Ji [25]. This procedure uses spectral decomposition of the observed correlation between SNPs to estimate the number of completely and partially correlated tests. Thus, to control for type I error, the nominal significance level of 0.05 is adjusted by the estimated effective number of tests using the normal Bonferroni procedure. The spectral decomposition was performed using modified scripts downloaded from the website of Dale Nyholt (http://gump.qimr.edu.au/general/daleN/SNPSpD, 4 July 2005), along with GOLD 1.1.0 [26] and R 2.7.0 (http://www.R-project.org).

## Results

**Figure 2:**
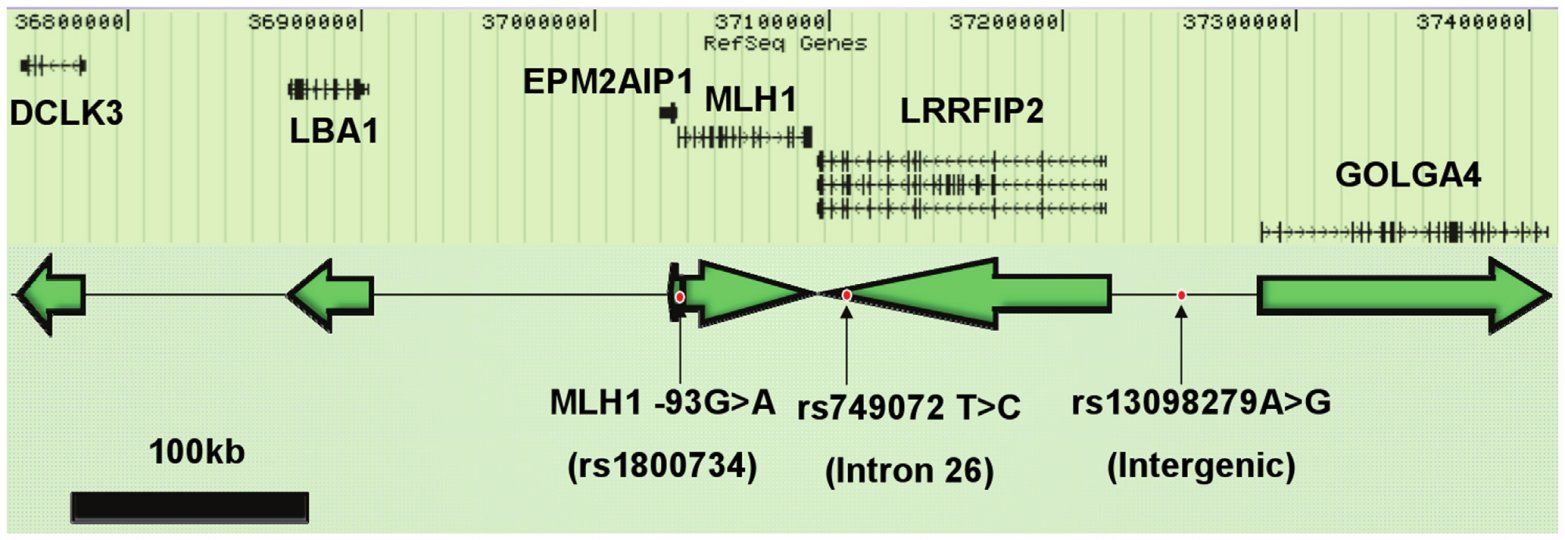
Region of chromosome 3 examined with genes and 3 SNPs. A total of 99 polymorphisms were examined in the Ontario samples across a 500kb region of chromosome 3 surrounding the *MLH1* gene. Genes in this region are outlined (top panel) along with their transcriptional directionality (bottom panel). The three polymorphisms of interest are indicated. Modified from Ensembl (www.ensembl.org).

We genotyped 901 cases and 1097 controls from Ontario for 99 SNPs in a 500 kb region of chromosome 3 surrounding the *MLH1* gene (**Figure 2**).

We removed 25 SNPs due to quality control issues: minor allele frequency <1% (22), call rate <87% (1), or Hardy-Weinberg *P*-value <10^−4^ (2), resulting in 74 analyzed SNPs. We then screened the Newfoundland (479 cases and 336 controls) and Seattle (591 cases and 629 controls) samples for 19 and 16 SNPs of interest, respectively. All markers in the Newfoundland and Seattle samples passed quality control filters. Tumor microsatellite instability was evaluated for 744 Ontario, 463 Newfoundland, and 487 Seattle cases. MLH1 IHC staining was undertaken on 709 Ontario, 462 Newfoundland, and 517 Seattle cases, and *MLH1* promoter methylation analysis was performed on 569 Ontario, 468 Newfoundland, and 210 Seattle cases. Characteristics of all three sample populations are summarized in **Table 1**. General clinical and pathologic features of CRC of our total case populations were similar to those of case populations used in the multiple logistic regression models, with the exception of Seattle, where there was a bias towards MSI-H tumors (and correspondingly IHC-deficient tumors). The list of all polymorphisms genotyped is provided in **Supplementary File S1**. Spectral decomposition revealed that testing the 74 SNPs in the Ontario samples was equivalent to 28 effective tests; hence, the association *P*-values were compared to a critical threshold of *P* = 0.0018, to control the experiment-wise significance level to 5%. For the Newfoundland data, analysis of the 19 SNPs constituted 8 effective tests (*P*-value threshold  = 0.0063), and for the Seattle data the 16 SNPs was equivalent to 6 effective tests (*P*-value threshold  = 0.0083).

**Table 1:** Characteristics of Study Populations.

All Subjects	Ontario	Seattle	Newfoundland
Controls No.	1097	629	336
Percent male	56	41	55
Age at exam, y – mean (sd)	64.3 (8.6)	60.6 (10.2)	60.2 (8.6)
Cases No.	901	591	479
Percent male	53	40	62
Age at exam, y – mean (sd)	61.6 (9.0)	60.1 (10.2)	62.3 (9.1)
Age at diagnosis, y – mean (sd)	60.7 (9.0)	60.1 (10.2)	60.9 (8.9)
Tumor Histological Grade No.	719	541	417
1 – Well differentiated – No. (%)	79 (11.0)	46 (8.5)	58 (13.9)
2 – Moderately differentiated – No. (%)	552 (76.8)	374 (69.1)	324 (77.7)
3 – Poorly differentiated – No. (%)	88 (12.2)	121 (22.3)	35 (8.4)
TNM Stage No.	751	499	Na
Stage I – No. (%)	172 (22.9)	150 (30.1)	Na
Stage II – No. (%)	291 (38.7)	140 (28.1)	Na
Stage III – No. (%)	241 (32.1)	167 (33.5)	Na
Stage IV – No. (%)	47 (6.3)	42 (8.4)	Na
Tumor MSI status, No.	744	487	463
MSI-high – No. (%)	90 (12.1)	75 (15.4)	40 (8.6)
MSI-low – No. (%)	3 (0.4)	48 (9.9)	23 (5.0)
MSI-stable – No. (%)	651 (87.5)	364 (74.7)	400 (86.4)
MLH1 IHC status, No.	709	517	462
IHC present – No. (%)	635 (89.6)	447 (86.5)	428 (92.6)
IHC deficient – No. (%)	74 (10.4)	70 (13.5)	34 (7.4)
*MLH1* promoter methylation status, No.	569	210	468
Methylation positive – No. (%)	62 (10.9)	58 (27.6)	25 (5.3)
Methylation negative – No. (%)	507 (89.1)	152 (72.4)	443 (94.7)
**Subjects With No Missing Data For the Variables Used in the Multiple Logistic Regression Models**			
Controls No.	1097	628	330
Percent male	56	41	55
Age at exam, y – mean (sd)	64.3 (8.6)	60.6 (10.2)	60.2 (8.6)
Cases No.	526	193	457
Percent male	52	32	62
Age at diagnosis, y – mean (sd)	60.9 (8.7)	60.5 (10.2)	61.0 (8.9)
Tumor Histological Grade, No.	471	188	402
1 – Well differentiated – No. (%)	49 (10.4)	14 (7.4)	57 (14.2)
2 – Moderately differentiated – No. (%)	363 (77.1)	122 (64.9)	310 (77.1)
3 – Poorly differentiated – No. (%)	59 (12.5)	52 (27.7)	35 (8.7)
Tumor TNM Stage, No.	488	179	Na
Stage I – No. (%)	105 (22.1)	41 (22.9)	Na
Stage II – No. (%)	194 (39.8)	59 (33.0)	Na
Stage III – No. (%)	161 (33.0)	70 (39.1)	Na
Stage IV – No. (%)	28 (5.7)	9 (5.0)	Na
Tumor MSI status, No.	526	193	457
MSI high – No. (%)	71 (13.5)	67 (34.7)	40 (8.8)
MSI low – No. (%)	1 (0.2)	41 (21.2)	22 (4.8)
MSI-stable – No. (%)	454 (86.3)	85 (44.0)	395 (86.4)
MLH1 IHC status, No.	526	193	457
IHC present – No. (%)	464 (88.2)	131 (67.9)	423 (92.6)
IHC deficient – No. (%)	62 (11.8)	62 (32.1)	34 (7.4)
*MLH1* promoter methylation status, No.	526	193	457
Methylation positive – No. (%)	54 (10.3)	56 (29.0)	25 (5.5)
Methylation negative – No. (%)	472 (89.7)	137 (71.0)	432 (94.5)

Na  =  not available, TNM  =  tumor-node-metastasis, IHC  =  immunohistochemistry, MSI  =  microsatellite instability, y  =  year, sd  =  standard deviation.

We first tested for association between each SNP and the risk of CRC (vs. controls), MSI-H CRCs (vs. MSS/L CRCs), MLH1 IHC-deficient CRCs (vs. MLH1 IHC-positive), and with *MLH1* promoter methylation (vs. unmethylated *MLH1* promoter) (**Supplementary File S2**). Two SNPs were statistically significantly associated with increased risk of CRC in Ontario: rs931913 (*P* = 0.001) and rs4624519 (*P* = 0.005).

Three additional SNPs were significantly associated with increased risk of MSI-H CRCs, MLH1 IHC-deficient CRCs, and with *MLH1* promoter methylated CRCs in Ontario (for rs1800734 *P* = 0.005, *P* = 0.04, and *P* = 0.018 respectively; for rs749072 *P* = 3.0×10^−4^, *P* = 0.011, and *P* = 0.003 respectively; and for rs13098279 *P* = 0.017, *P* = 0.090, and *P* = 0.037 respectively; **Supplementary File S2**). We examined these findings in the two other samples: for rs1800734 in Newfoundland, *P* = 8.53×10^−5^, 1.92×10^−5^, and 8.95×10^−7^ for MSI-H, MLH1 IHC-deficiency, and *MLH1* promoter methylation respectively and, for Seattle, *P* = 0.08, *P* = 0.02, and *P* = 0.04 respectively; for rs749072 in Newfoundland, *P* = 0.001, *P* = 2.4×10^−4^, *P* = 6.65×10^−6^ respectively and, for Seattle, *P* = 0.03, *P* = 0.004, and *P* = 0.014 respectively; for rs13098279 in Newfoundland, *P* = 4.5×10^−4^, *P* = 4.30×10^−5^, and 1.98×10^−6^ respectively and, for Seattle, *P* = 0.24, *P* = 0.07, and *P* = 0.14 respectively. See **Supplementary File S2**. None of the three latter SNPs were significantly associated with overall risk of CRC in the three samples studied (**Supplementary File S2**). These three SNPs span a 197.5-kb region with rs1800734 located in the *MLH1* promoter, 93 nucleotides upstream of the translational start site; rs749072 located in intron 26 of *LRRFIP2* (IVS26-18T>C); and rs13098279 located between *LRRFIP2* and *GOLGA4* (**Figure 2**). All three SNPs are in strong linkage disequilibrium in the Ontario controls (pairwise *r^2^* >0.73, *D*' >0.98). Pairwise *D*' and *r^2^* for all SNPs genotyped in Ontario control subjects are shown in **Supplementary Figures S1 and S2**.

Analysis of all three samples combined revealed strong associations between rs749072 and decreased risk of *MLH1*-promoter-methylated CRC (*P* = 3.80×10^−6^, OR for the common allele  = 0.45, CI = 0.34–0.60); increased risk of MLH1-protein-expressing CRC as measured by IHC staining (*P* = 3.99×10^−7^, OR for the common allele  = 1.87, CI = 1.47–2.39); and decreased risk of MSI-H CRC (*P* = 2.50×10^−7^, OR for the common allele  = 0.55, CI = 0.44–0.69). Because the other two SNPs (rs1800734 and rs13098279) are in strong linkage disequilibrium with rs749072, analyses of these SNPs yielded similar results (**Table 2**).

**Table 2:** Single marker analysis in the combined data for 3 SNPs for CRC cases versus controls, *MLH1* promoter methylation, MLH1 IHC staining and MSI tumor status.

Colon Cancer Cases vs. Controls						
Marker	Common Allele	Sample Size	*P*-value	OR	Lower 95% CI	Upper 95% CI
rs1800734	G	3923	0.108	0.915	0.822	1.020
rs749072	T	3912	0.102	0.918	0.828	1.017
rs13098279	G	3912	0.155	0.924	0.828	1.031

*MLH1* Promoter Methylation within cases (positive versus negative)						
Marker	Common Allele	Sample Size	*P*-value	OR	Lower 95% CI	Upper 95% CI
rs1800734	G	1236	3.25E-07	0.470	0.352	0.628
rs749072	T	1202	3.80E-06	0.451	0.340	0.599
rs13098279	G	1202	5.80E-06	0.508	0.379	0.681

MLH1 IHC Staining within cases (positive versus negative)						
Marker	Common Allele	Sample Size	*P*-value	OR	Lower 95% CI	Upper 95% CI
rs1800734	G	1674	2.43E-06	1.823	1.420	2.339
rs749072	T	1640	3.99E-07	1.872	1.469	2.386
rs13098279	G	1640	4.71E-05	1.691	1.313	2.179
rs13098279	G	1648	1.15E-04	0.626	0.494	0.794

Tumor MSI Status within cases (MSI-H versus MSS/L)						
Marker	Common Allele	Sample Size	*P*-value	OR	Lower 95% CI	Upper 95% CI
rs1800734	G	1681	3.38E-06	0.574	0.454	0.725
rs749072	T	1648	2.50E-07	0.550	0.439	0.691

Analyses of CRC cases versus controls are adjusted for age, sex, and site.

OR  =  odds ratio, CI  =  confidence interval.

Single marker results for the above SNPs for each study population are shown in **Supplementary File S2**.

In order to examine whether these SNPs were associated with the pathway that we hypothesized (**Figure 1**), we next created logistic regression models for MSI-H versus non-MSI-H CRCs for the combined dataset (**Supplementary File S3**). We modelled MSI-H as a function of each of the upstream predictors, as well as combinations of predictors: first MLH1 IHC status; then *MLH1*-promoter-methylation status; a SNP; both MLH1 IHC status and *MLH1* promoter methylation status; and finally MLH1 IHC status, *MLH1*-promoter-methylation status and each SNP (**Table 3**). MLH1 IHC status alone was a strong predictor of MSI-H CRCs (*P* = 2.08×10^−30^) as was the *MLH1*-promoter-methylation status (*P* = 1.33×10^−44^) for the SNPs of interest (for rs1800734, *P* = 2.30×10^−4^, for rs749072 *P* = 1.36×10^−5^, and for rs13098279 *P* = 5.10×10^−3^). The model with MLH1 IHC status and *MLH1*-promoter-methylation status gave the smallest Akaike's Information Criterion (AIC) (225.12) and addition of rs13098279 resulted in the second most parsimonious model (AIC = 225.94) (**Table 3**). In the model with MLH1 IHC status and *MLH1*-promoter-methylation status, both variables were statistically significant, as was the SNP in the model where it was the sole predictor. However, when the SNP of interest was added to the model with MLH1 IHC status and *MLH1*-promoter-methylation status, the SNP no longer remained statistically significant: the *P*-value from the test of significance for rs1800734 changed from 2.30×10^−4^ when it was the sole predictor, to 0.72 when the SNP, *MLH1* promoter methylation status and MLH1 IHC status were predictors; for rs749072, from 1.36×10^−5^ to 0.98; and for rs13098279, from 0.005 to 0.29 (**Table 3** **)**. In the most parsimonious model, recruitment centre did not have a significant effect on the model (*P* ≥0.26, **Supplementary File S3**).

**Table 3:** Logistic regression model results for MSI status with various predictor combinations in the combined data.

Model No.	Covariate	AIC	Parameter Estimate	Standard Error	*P*-value
1	IHC	238.72	7.79	0.68	2.08E-30
2	CH3	470.64	5.56	0.40	1.33E-44
3	IHC	225.12	6.53	0.68	7.36E-22
	CH3		3.03	0.66	4.29E-06
4	rs1800734	890.89	−0.49	0.13	2.30E-04
5	IHC	240.83	7.74	0.68	2.31E-30
	rs1800734		−0.03	0.33	0.94
6	CH3	472.63	5.55	0.40	1.52E-43
	rs1800734		0.04	0.22	0.85
7	IHC	227.05	6.50	0.67	5.98E-22
	CH3		3.06	0.67	5.18E-06
	rs1800734		0.12	0.34	0.72
4	rs749072	885.46	−0.56	0.13	1.36E-05
5	IHC	240.72	7.72	0.67	2.70E-30
	rs749072		−0.13	0.31	0.68
6	CH3	472.56	5.52	0.40	2.24E-43
	rs749072		−0.08	0.21	0.70
7	IHC	227.23	6.48	0.67	5.64E-22
	CH3		3.01	0.66	5.72E-06
	rs749072		−0.01	0.32	0.98
4	rs13098279	896.57	−0.38	0.14	0.0051
5	IHC	240.41	7.80	0.68	2.73E-30
	rs13098279		0.21	0.35	0.55
6	CH3	471.93	5.60	0.40	1.23E-43
	rs13098279		0.19	0.23	0.41
7	IHC	225.94	6.55	0.68	1.04E-21
	CH3		3.17	0.69	3.74E-06
	rs13098279		0.39	0.37	0.29

Age at diagnosis, sex, and location are covariates common to all the models described above. IHC refers to the MLH1 immunohistochemical staining variable, CH3 refers to the *MLH1* promoter methylation variable, AIC  =  Akaike's information criterion. Logistic regression models for each SNP per study population and for the combined data are shown in **Supplementary File S3**.

The role of three SNPs of interest, rs1800734, rs749072, and rs13098279, is explored.

We evaluated the same models in the location-specific datasets and the results were consistent with the combined results (**Supplementary File S3**). MLH1 IHC status, *MLH1* promoter methylation status, and the SNPs of interest were all strong predictors of tumor MSI-H status. The model that included MLH1 IHC status and *MLH1*-promoter-methylation status gave the smallest AIC in all three samples. The addition of any of the three SNPs did not result in a significantly better model fit (**Supplementary File S3)**.

## Discussion

This large-scale multi-center study examined germline DNA markers and their contributions to somatic events, especially susceptibility to DNA methylation in CRC. In three independent samples, three polymorphisms, rs1800734, rs749072, and rs13098279 were associated with *MLH1*-promoter-methylation status resulting in loss of MLH1 protein and microsatellite instability. Although these three markers are not associated with an increase in the risk of CRC overall, they do play a role in colorectal tumorigenesis in the subset of CRCs that display genome-wide microsatellite instability. Among cases in each individual sample population and in an analysis of all three combined, statistically significant associations were observed between each of these three polymorphisms and *MLH1* promoter methylation, MLH1 IHC deficiency, and MSI-H tumor status. In multiple logistic regression models, each SNP was associated with tumor MSI-H status; however, once MLH1 IHC deficiency or *MLH1* promoter methylation, or both, were included in the model, the SNP association was no longer statistically significant. The observation that the SNP term was not significant in the model with MLH1 IHC and *MLH1* promoter methylation indicates that the addition of the SNP does not significantly improve model fit over and above what MLH1 IHC and *MLH1* methylation contribute to the model. Hence, MSI status is conditionally independent of the SNP, or in other words, the effect of the SNP on MSI status is contained in the effects of MLH1 IHC and *MLH1* methylation on MSI. These results support the hypothesis that the observed associations between these polymorphisms and MSI-H status occur through *MLH1* methylation and subsequent gene silencing. Furthermore, when both IHC and methylation status were included in the model, MLH1 IHC status and *MLH1* promoter methylation were both strongly associated with MSI-H status indicating that these two events, while highly correlated, are not completely dependent on each other even after exclusion of all known germline MMR gene mutation carriers. A similar observation was reported previously where *MLH1* promoter methylation accounted for 80% of MLH1 IHC-deficient-MSI-H CRCs after excluding all *MLH1* germline mutation carriers [27]. Other mechanisms must, then, be responsible for the remaining 20% of MLH1 IHC-deficient-MSI-H CRCs. These may include somatic gene mutations, epimutations, loss of heterozygosity at an MMR gene locus, or maybe even unidentified microRNA silencing of a MMR gene.

In addition to colon cancer, the *MLH1*-93G>A polymorphism (rs1800734) also is associated with other cancers including: ovarian [28], endometrial [10], [29], and secondary tumors arising from Hodgkin lymphoma [30]. More specifically, the *MLH1*-93G>A polymorphism was shown to be associated with *MLH1* promoter methylation in endometrial cancers [10]. Hodgkin lymphoma patients who carried the variant -93A allele were at higher risk of developing secondary tumors following methylating chemotherapy [30]. In the colon, this polymorphism has been shown to increase the risk of hyperplastic polyps and adenomas in smokers [31] as well as MSI-H CRCs, alone, or in combination with lifestyle factors [32]. Furthermore, the *MLH1*-93G>A polymorphism is associated with CIMP-positive CRCs (which include *MLH1* promoter methylation) [9] and with the loss of *MLH1* gene expression [11], both of which are consistent with the hypothesis proposed and tested here.

One possible explanation of our previous finding that the *MLH1*-93G>A promoter polymorphism was associated with increased risk of MSI-H CRCs is that the association is caused by another functional *MLH1* polymorphism in strong linkage disequilibrium (LD) with the *MLH1*-93G>A SNP [8]. In this study, we identified two polymorphisms, rs749072 and rs13098279, that are in strong LD with the *MLH1*-93G>A SNP. However, neither of these two polymorphisms are located in *MLH1*: rs749072 is located in intron 26 of *LRRFIP2* (leucine-rich repeat in Flightless interaction protein 2), 18 nucleotides from a splice acceptor site (IVS26-18T>C); rs13098279 is an intergenic polymorphism located between the *LRRFIP2* and *GOLGA4* (golgi autoantigen, golgin subfamily a, 4). LRRFIP2 binds Dishevelled and serves as an activator of the Wnt signalling pathway, which is deregulated in ∼85% of CRCs [33]. LRRFIP2 splice variants were identified in colon and prostate cancers [34]. The spliced exons contain several potential phosphorylation sites that might influence protein function [34]. The roles of the identified splice variants in tumorigenesis, as well as potential effects of rs749072 on splicing machinery, are still unclear.

We identified two additional polymorphisms, rs931913 and rs4624519, associated with an overall increased risk of CRC in the Ontario sample. We did not attempt to replicate the findings for rs931913 and rs4624519 in Newfoundland or Seattle.

Our study has several limitations, including the unavailability of some clinical data from our study subjects. Clinical and pathologic characteristics were not available for several reasons (e.g., tumor material not available for MSI, IHC, or methylation testing, technical difficulties, or death of the patient before tissue samples could be obtained). However, because the general clinical and pathologic characteristics of CRC in our whole population were similar to those of cases with no missing data, our study was not limited by this potential source of bias. One exception was the methylation analysis of Seattle samples, which were mostly completed on MSI-H cases. However, the results obtained from the Seattle samples are very similar to those from Ontario and Newfoundland.

Our study also has numerous strengths. The large sample size gave us high power and precision. In order to observe statistically significant associations of the same order of magnitude that we report here in a genome-wide association study design, we would require between 23,000 and 61,000 cases and controls. A major strength of our study is the use of three independent population-based registries, Ontario, Newfoundland, and Seattle. Replication of our main findings in two additional independent samples provides strong evidence that our findings reflect real associations and are unlikely to have occurred by chance.

The important finding of this study is the identification of a genetic basis for DNA methylation susceptibility; it indicates that genetic variants may play an indirect role in increasing the risk of MSI-H colorectal cancer. Perhaps they alter the binding sites of transcription factors and DNA-binding proteins that protect the DNA molecule from methylation. Inability of these protective proteins to bind DNA would expose DNA to methylating machinery. Conversely, these polymorphisms may create binding sites for co-repressors, methylated DNA-binding proteins, or other proteins involved in epigenetic silencing that modify DNA and silence gene expression. Another possible mechanism involves the production of antisense RNA; it was shown recently that increased production of antisense RNA resulted in epigenetic silencing of p15 tumor suppressor gene [35]. The polymorphisms in this study may increase the production of antisense RNAs that result in epigenetic silencing of the corresponding sense-strand genes.

The fact that polymorphisms in genes other than *MLH1* are associated with DNA methylation may indicate that the *MLH1* promoter methylation observed in MSI-H colorectal cancers is not localized just to the *MLH1* locus, but extends beyond the gene. Indeed, Hitchins *et al*. observed that, in MSI-H colorectal cancers, methylation is not limited to the *MLH1* promoter region, but affects genes in a region as large as 2.4 Mega base-pairs [36]. We may have identified, in a much smaller region, genetic markers of the predisposition to such epigenetic alterations and, because a mismatch repair gene, *MLH1*, is involved, microsatellite instability invariably develops. However, we cannot yet exclude the possibility that these markers tag some other unknown variant(s) that are the true cause of DNA susceptibility to methylation.

The major agent used for the medical treatment of patients with advanced CRC, 5-Fluorouracil (5-FU), is recognized by the MMR system [37]. 5-FU selectively kills cells with intact MMR, while MMR-deficient cells are resistant [37]. Patients with stage II and III sporadic MSI CRC do not show a survival benefit following 5-FU therapy when compared with MSS CRC patients in retrospective and prospective studies [38], [39], [40]. Indeed, 5-FU-based adjuvant chemotherapy might decrease overall and disease-free survival among MSI CRC patients [38]. Similarly, stage III Lynch Syndrome patients do not show a 5-year survival benefit with 5-FU treatment over untreated patients [41]. CRC is a heterogeneous disease and the three polymorphisms used in this study may serve as predictive markers in at-risk individuals for early identification of MSI and selection of optimal therapies.

In summary, we built on our previous finding, an association of the *MLH1*-93G>A polymorphism with MSI-H colorectal cancers [8]. We identified a novel mechanism in which common missense alterations may contribute to complex disease. The three polymorphisms reported in this study serve as germline markers/predisposition alleles for a somatic event that will result in gene silencing and consequently, a specific subtype of colorectal cancer. Additional characterization of these the genes and polymorphisms noted here may lead to new insights and new mechanisms by which alleles contribute to cancer incidence and progression.

### Supporting Information

File S1 List of all SNPs genotyped in Ontario, Newfoundland and Seattle samples.
(0.06 MB XLS)

File S2 Analyses of all SNPs with CRC, tumor MSI status, MLH1 IHC.
(0.15 MB XLS)

File S3 Information on all statistical models used.
(0.15 MB XLS)

File S4 Contains supplementary Table S1: Sequences of primers and probes F  =  forward primer; R  =  reverse primer; FAM  =  wild type allele probe; VIC  =  variant allele probe; MGBNFQ  =  minor groove binder non-florescent quencher, FM  =  methylated forward primer, RM  =  methylated reverse primer, FU - unmethylated forward primer, RU  =  unmethylated reverse primer, BHQ-1  =  black hole quencher-1. *Published previously (23).
(0.03 MB DOC)

Figure S1 D-Prime map of all SNPs genotyped in Ontario samples.
(0.11 MB JPG)

Figure S2 R-squared map of all SNPs genotyped in Ontario samples.
(0.14 MB JPG)
