# Supplementary material for: Specific Variants in the MLH1 Gene Region May Drive DNA Methylation, Loss of Protein Expression, and MSI-H Colorectal Cancer
Source: PLoS One. 2010 Oct 13;5(10):e13314. doi: 10.1371/journal.pone.0013314 (PMC2954166; doi:10.1371/journal.pone.0013314)
Supplement: File S4 — Contains supplementary Table S1: Sequences of primers and probes F = forward primer; R = reverse primer; FAM = wild type allele probe; VIC = variant allele probe; MGBNFQ = minor groove binder non-florescent quencher, FM = methylated forward primer, RM = methylated reverse primer, FU - unmethylated forward primer, RU = unmethylated reverse primer, BHQ-1 = black hole quencher-1. *Published previously (23). (0.03 MB DOC) [file pone.0013314.s004.doc]

Supplementary Table S1: Sequences of primers and probes

| Gene/SNP | Primer**/**Probe Sequences |
| --- | --- |
| LRRFIP2  IVS26-18T>C  rs749072 | F – ACCAAAAAGTGGTGACTTCTAGTGC  R – CTCAAGCCGGCTAATCTGTAAGTAT  FAM – ACTAGAGGCCTATGTTCT-MGBNFQ  VIC – TACTAGAGGCCTGTGTTCT-MGBNFQ |
| rs13098279 | F – TGCAGCTTGAGTTGACAAATGAAA  R – TAAATCCTGCTTCATCAGCTGAGG  FAM – TAAGACATCTCACCATAAGGA-MGBNFQ  VIC – ACATAAGACATCTTACCATAAG-MGBNFQ |
| LBA1 rs4431050  IVS7+477G>A | F – TGGAGTACACGTACTGGAGGTAGA  R – CCCAGGGATCTGGATGTGTT  FAM – TGATACCATCTCTATCCCC-MGBNFQ  VIC – TGATACCATCTTTATCCCC-MGBNFQ |
| MLH1 MethyLight* | F – AGGAAGAGCGGATAGCGATTT  R – TCTTCGTCCCTCCCTAAAACG  FAM – CCCGCTACCTAAAAAAATATACGCTTACGCG-BHQ-1 |
| Alu-C4 MethyLight* | F – GGTTAGGTATAGTGGTTTATATTTGTAATTTTAGTA  R – ATTAACTAAACTAATCTTAAACTCCTAACCTCA  FAM – CCTACCTTAACCTCCC - MGBNFQ |

F = forward primer; R = reverse primer; FAM = wild type allele probe; VIC = variant allele probe; MGBNFQ = minor groove binder non-florescent quencher, FM = methylated forward primer, RM = methylated reverse primer, FU – unmethylated forward primer, RU = unmethylated reverse primer, BHQ-1 = black hole quencher-1.

*Published previously (23).
