# Supplementary figures and images for: Specific Variants in the MLH1 Gene Region May Drive DNA Methylation, Loss of Protein Expression, and MSI-H Colorectal Cancer
Source: PLoS One. 2010 Oct 13;5(10):e13314. doi: 10.1371/journal.pone.0013314 (PMC2954166; doi:10.1371/journal.pone.0013314)

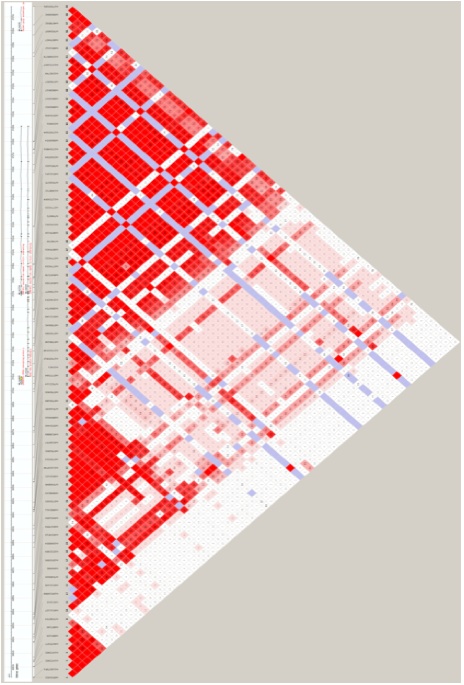

Supplement: Figure S1 — D-Prime map of all SNPs genotyped in Ontario samples. (0.11 MB JPG) [file pone.0013314.s005.jpg]

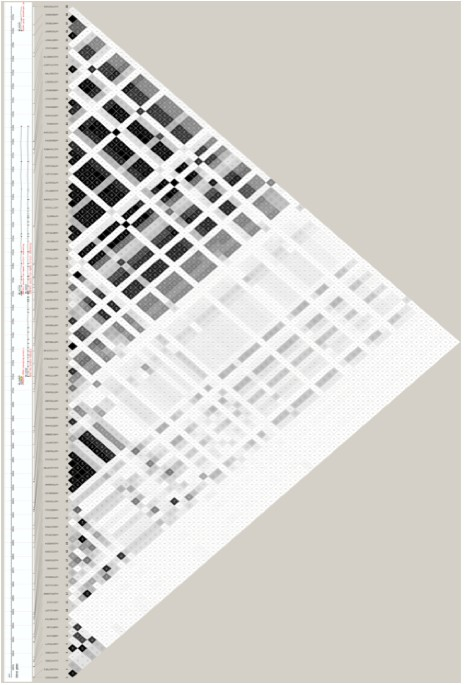

Supplement: Figure S2 — R-squared map of all SNPs genotyped in Ontario samples. (0.14 MB JPG) [file pone.0013314.s006.jpg]
